# Predictors of Postoperative Complications After Retromuscular Incisional Hernia Repair: A Retrospective Cohort Study

**DOI:** 10.3390/jcm15082935

**Published:** 2026-04-12

**Authors:** Daniel Ioan Mihalache, Niculae Iordache, Liviu Vasile, Stelian-Stefaniță Mogoantă, Tiberiu-Ștefăniță Țenea-Cojan, Nicolae-Dragoș Mărgăritescu, Laurențiu Augustus Barbu

**Affiliations:** 1 Doctoral School, “Carol Davila” University of Medicine and Pharmacy, 37 Dionisie Lupu Street, 020021 Bucharest, Romania; dan64mm@yahoo.com; 2 Department of Surgery, “Carol Davila” University of Medicine and Pharmacy, 37 Dionisie Lupu Street, 020021 Bucharest, Romania; niordache@gmail.com; 3 Department of Surgery, Emergency County Hospital, University of Medicine and Pharmacy of Craiova, 2 Petru Rares Street, 200349 Craiova, Romania; ssmogo@yahoo.com (S.-S.M.); dmargaritescu@yahoo.com (N.-D.M.); 4 Department of Surgery, Railway Clinical Hospital Craiova, University of Medicine and Pharmacy of Craiova, 2 Petru Rares Street, 200349 Craiova, Romania; tiberiu.tenea@umfcv.ro (T.-Ș.Ț.-C.); laurentiu.barbu@umfcv.ro (L.A.B.)

**Keywords:** incisional hernia, retromuscular repair, Rives–Stoppa technique, abdominal wall reconstruction, postoperative complications, risk factors

## Abstract

**Background**: Incisional hernias are a frequent complication after abdominal surgery and may significantly affect patient outcomes. Retromuscular mesh placement using the Rives–Stoppa technique is widely considered a reliable approach for abdominal wall reconstruction, although postoperative complications remain an important clinical concern. Identifying predictors of adverse outcomes may improve patient selection and perioperative management. **Methods**: This retrospective cohort study included 1262 patients who underwent retromuscular incisional hernia repair. Demographic characteristics, comorbidities, hernia features, operative data, and postoperative outcomes were analyzed. Univariate and multivariable logistic regression analyses were performed to identify independent predictors of postoperative complications. Model performance was evaluated using receiver operating characteristic analysis. **Results**: The study included 1262 patients with a mean age of 61.5 ± 12.4 years, with a slight predominance of women (55%). The overall complication rate was 19.5%, with seroma (10.5%), surgical site infection (7.0%), and hematoma (3.5%) being the most common events. Hernia recurrence occurred in 6.0% of patients during follow-up. Multivariable analysis identified obesity (*p* < 0.001), large defect size (W3) (*p* < 0.001), diabetes mellitus (*p* = 0.004), recurrent hernia (*p* = 0.013), and ASA III–IV status (*p* = 0.038) as independent predictors of postoperative complications. The predictive model demonstrated moderate discrimination (AUC ≈ 0.73). **Conclusions**: Retromuscular incisional hernia repair is associated with acceptable morbidity and low recurrence rates. Obesity (OR 2.41), large defect size (W3) (OR 2.12), diabetes mellitus (OR 1.89), recurrent hernia (OR 1.67), and American Society of Anesthesiologists (ASA) classification III–IV status (OR 1.54) were identified as independent predictors of postoperative complications. The predictive model demonstrated moderate discrimination (AUC ≈ 0.73), supporting its potential role in clinical risk stratification and perioperative decision-making.

## 1. Introduction

Incisional hernia represents one of the most frequent long-term complications following abdominal surgery and remains a major challenge in abdominal wall reconstruction. The reported incidence ranges from approximately 10% to 30% after laparotomy, and these defects may lead to chronic pain, functional impairment, and reduced quality of life, often requiring surgical repair [1,2,3,4].

Over the past decades, prosthetic mesh reinforcement has become the standard approach in ventral and incisional hernia repair due to significantly lower recurrence rates compared with primary suture repair [5]. In particular, extraperitoneal mesh placement techniques have gained increasing attention, as they allow adequate defect coverage while minimizing contact with intra-abdominal viscera [1,6].

Among open surgical techniques, retromuscular repair as described by Rives and Stoppa is widely accepted for incisional hernia reconstruction. This approach involves restoration of the linea alba and placement of a mesh in the retrorectus space, providing a well-vascularized environment that supports mesh integration and reduces infection risk [7,8,9,10]. Several studies have reported favorable long-term outcomes associated with this technique [11,12,13].

Despite these advantages, postoperative complications remain an important concern. Surgical site occurrences such as seroma, hematoma, and infection may negatively affect recovery and long-term outcomes. Both patient-related factors (e.g., obesity, diabetes, smoking) and hernia-related factors (e.g., defect size, recurrence) have been shown to influence postoperative morbidity.

Recent advances in minimally invasive surgery, including laparoscopic and robotic retromuscular approaches, have further expanded treatment options. Techniques such as enhanced-view totally extraperitoneal (eTEP) repair aim to combine the biomechanical advantages of retromuscular mesh placement with the benefits of minimally invasive surgery [14,15]. However, the optimal surgical strategy and predictors of postoperative outcomes remain subjects of ongoing investigation.

Although numerous studies have evaluated ventral and incisional hernia repair, many include relatively small cohorts or heterogeneous techniques. Therefore, large contemporary clinical analyses are needed to better define predictors of postoperative complications and improve risk stratification [16,17].

The aim of the present study was to evaluate postoperative outcomes following retromuscular incisional hernia repair and to identify independent predictors of postoperative complications in a large cohort of patients.

## 2. Materials and Methods

### 2.1. Study Design and Setting

This retrospective cohort study included patients who underwent surgical repair of incisional hernias using the retromuscular Rives–Stoppa technique. All procedures were performed at the Emergency County Hospital of Ploiești, Romania, between January 2013 and December 2022.

A total of 1262 consecutive patients were included in the analysis. The study evaluated demographic characteristics, comorbidities, hernia features, operative parameters, and postoperative outcomes in order to identify predictors of postoperative complications following retromuscular incisional hernia repair.

### 2.2. Ethical Approval

The study protocol was reviewed and approved by the Ethics Committee of the Emergency County Hospital of Ploiești. Ethical approval was granted following evaluation of the research proposal submitted to the institutional ethics board (approval decision no. 13741/24 March 2023).

The study was conducted in accordance with the principles of the Declaration of Helsinki and institutional regulations regarding research involving human subjects. All patient data were anonymized prior to analysis and handled in accordance with the General Data Protection Regulation (GDPR).

Due to the retrospective nature of the study and the use of anonymized data, the requirement for individual informed consent was waived.

### 2.3. Patient Selection

All adult patients who underwent elective surgical repair of incisional hernias using the retromuscular Rives–Stoppa technique during the study period were eligible for inclusion. Patients undergoing repairs with component separation techniques were not included in this study.

#### 2.3.1. Inclusion Criteria

▪ Age ≥ 18 years;
▪ Diagnosis of incisional hernia following previous abdominal surgery;
▪ Elective open retromuscular repair using the Rives–Stoppa technique;
▪ Complete clinical and operative records available.

#### 2.3.2. Exclusion Criteria

▪ Primary ventral hernias;
▪ Emergency hernia repairs;
▪ Patients with incomplete medical records;
▪ Patients undergoing alternative mesh placements (onlay or intraperitoneal techniques).

**Figure 1:**
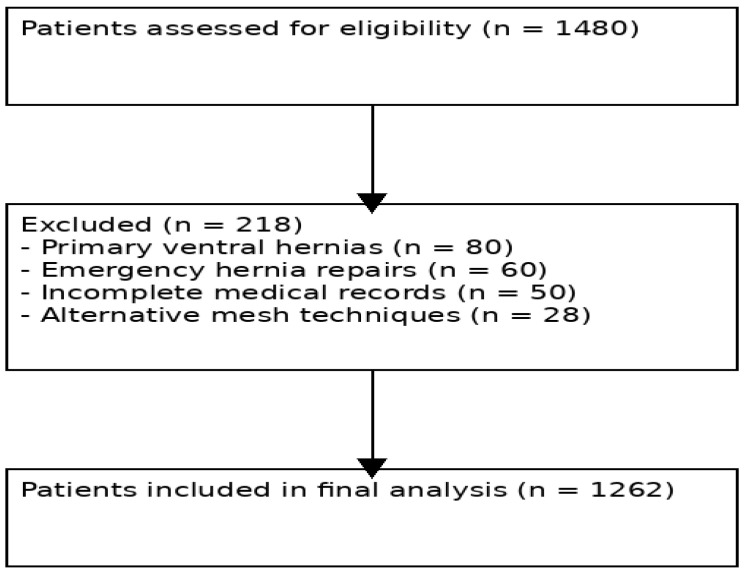
Flowchart of patient selection. The diagram illustrates the number of patients assessed for eligibility, excluded according to predefined criteria, and included in the final analysis.

A flowchart illustrating the patient selection process, including inclusion and exclusion criteria, has been added to improve transparency (Figure 1).

### 2.4. Surgical Technique

All procedures were performed using the open retromuscular repair technique described by Rives and Stoppa.

After midline laparotomy and adhesiolysis, the posterior rectus sheath was incised and the retrorectus space was developed bilaterally. The hernia sac was reduced, and the posterior fascial layer was reconstructed.

A synthetic mesh was then placed in the retromuscular plane with adequate overlap of the hernia defect. Finally, the anterior fascial layer was reconstructed to restore the integrity of the abdominal wall.

This technique allows mesh placement in a well-vascularized extraperitoneal space and provides tension-free reinforcement of the abdominal wall.

All procedures were performed by experienced surgeons following a standardized surgical protocol based on the Rives–Stoppa technique. Although minor variations were permitted depending on intraoperative findings, the key surgical principles were consistently applied in all cases.

No component separation technique, including unilateral or bilateral transversus abdominis release (TAR), was performed in this cohort. All repairs were limited to standard open retromuscular Rives–Stoppa reconstruction without posterior component separation.

### 2.5. Data Collection

Clinical data were collected retrospectively from hospital electronic medical records and operative reports.

The following variables were analyzed:

Patient-related variables:

▪ Age;
▪ Sex;
▪ Body mass index (BMI);
▪ Diabetes mellitus;
▪ Smoking status;
▪ Hypertension;
▪ Pulmonary disease (COPD or asthma);
▪ ASA classification.

Hernia-related variables:

▪ Hernia location (midline or lateral);
▪ Defect size according to the European Hernia Society (EHS) classification;
▪ Recurrent hernia at presentation.

Operative variables:

▪ Operative time;
▪ Length of postoperative hospital stay.

Postoperative outcomes:

▪ Seroma;
▪ Surgical site infection (SSI);
▪ Hematoma;
▪ Wound dehiscence;
▪ Mesh infection;
▪ Enterocutaneous fistula;
▪ Chronic postoperative pain;
▪ Hernia recurrence.

Patients with incomplete key clinical data were excluded from the analysis according to the predefined exclusion criteria, and no data imputation methods were applied.

### 2.6. Outcome Measures

The primary outcome of the study was the occurrence of postoperative complications following retromuscular incisional hernia repair. Secondary outcomes included individual postoperative complications, length of hospital stay, chronic postoperative pain, and hernia recurrence during follow-up.

Patients were followed postoperatively through clinical visits and review of hospital records to assess postoperative complications and recurrence. However, follow-up duration was not uniform across patients, and complete follow-up data were not available for all cases.

Postoperative complications were defined according to standard surgical definitions. Surgical site infection (SSI) was defined according to the Centers for Disease Control and Prevention (CDC) criteria. Seroma was defined as a clinically or radiologically detected fluid collection at the surgical site requiring observation, aspiration, or drainage. Chronic postoperative pain was defined as pain persisting for more than three months after surgery.

### 2.7. Statistical Analysis

Statistical analysis was performed to evaluate factors associated with postoperative complications following retromuscular incisional hernia repair. Continuous variables were assessed for normality using the Shapiro–Wilk test and are presented as means ± standard deviation (SD). Categorical variables are expressed as absolute frequencies and percentages.

Comparisons between patients with and without postoperative complications were performed using the chi-square test or Fisher’s exact test for categorical variables, as appropriate.

Univariate analysis was initially performed to explore associations between potential risk factors and postoperative complications. Variables showing statistical significance in univariate analysis were subsequently included in a multivariable logistic regression model to identify independent predictors of postoperative complications.

The results of logistic regression analyses are reported as odds ratios (OR) with 95% confidence intervals (CI).

Model calibration was evaluated using the Hosmer–Lemeshow goodness-of-fit test.

Receiver operating characteristic (ROC) curve analysis was performed to evaluate the discriminatory performance of the multivariable model for predicting postoperative complications. Model accuracy was assessed using the area under the curve (AUC). All statistical tests were two-tailed, and a *p*-value < 0.05 was considered statistically significant. Data were initially processed using Microsoft Excel 2016 (Microsoft Corp., Redmond, WA, USA) with XLSTAT 2022 (Addinsoft SARL, Paris, France), and statistical analyses were performed using SPSS version 27.0 (IBM Corp., Armonk, NY, USA).

## 3. Results

The baseline characteristics of the study population are presented in Table 1. A total of 1262 patients undergoing retromuscular incisional hernia repair were included. The mean age was 61.5 ± 12.4 years, and 55% of patients were female.

The mean body mass index was 31.2 ± 5.8 kg/m^2^. Obesity (BMI ≥ 30) was present in 52% of patients, while 33% were overweight.

Diabetes mellitus was present in 22% of patients, smoking in 20%, and hypertension in 62%. Pulmonary disease was reported in 11% of cases. Recurrent hernia at presentation was observed in 23% of patients. According to the American Society of Anesthesiologists (ASA) classification, 65% of patients were ASA I–II and 35% were ASA III–IV.

Hernia characteristics and operative data are presented in Table 2. Midline hernias accounted for 85% of cases, while 15% were lateral defects. According to the European Hernia Society (EHS) classification, 55% of defects were classified as W2 (4–10 cm), 25% as W1 (<4 cm), and 20% as W3 (>10 cm).

The mean operative time was 135 ± 35 min, and the mean postoperative hospital stay was 7.5 ± 3.2 days.

Postoperative complications are summarized in Table 3. The overall complication rate was 19.5%. Seroma occurred in 10.5% of patients, surgical site infection in 7.0%, and hematoma in 3.5%. Wound dehiscence and mesh infection were each reported in 1.5% of cases, while enterocutaneous fistula occurred in 0.5%.

Hernia recurrence was observed in 6.0% of patients during follow-up, and chronic postoperative pain was reported in 3.0% of cases.

In univariate analysis (Table 4), obesity (BMI ≥ 30) was associated with postoperative complications (*p* < 0.001). Diabetes mellitus (*p* = 0.012), smoking (*p* = 0.041), recurrent hernia (*p* = 0.008), large defect size (W3) (*p* < 0.001), and ASA III–IV classification (*p* = 0.015) were also significantly associated with postoperative complications.

Multivariable logistic regression analysis identified several independent predictors of postoperative complications (Table 5). Obesity (BMI ≥ 30) (OR 2.41, 95% CI 1.72–3.38, *p* < 0.001), large defect size (W3) (OR 2.12, 95% CI 1.41–3.19, *p* < 0.001), diabetes mellitus (OR 1.89, 95% CI 1.23–2.91, *p* = 0.004), recurrent hernia (OR 1.67, 95% CI 1.11–2.51, *p* = 0.013), and ASA III–IV classification (OR 1.54, 95% CI 1.02–2.31, *p* = 0.038) were independently associated with postoperative complications.

**Figure 2:**
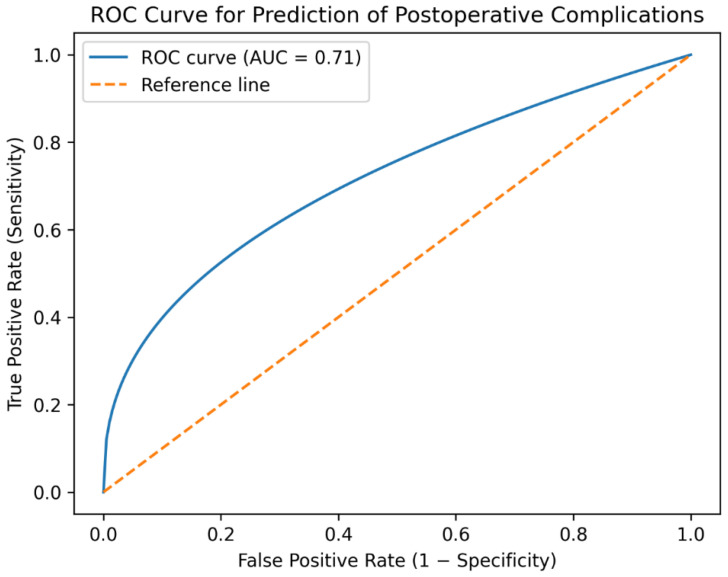
Receiver operating characteristic (ROC) curve of the multivariable logistic regression model predicting postoperative complications after retromuscular incisional hernia repair.

Receiver operating characteristic (ROC) analysis demonstrated a moderate discriminatory ability of the multivariable model for predicting postoperative complications, with an area under the curve (AUC) of 0.73 (Figure 2). The Hosmer–Lemeshow test indicated good model calibration (*p* > 0.05).

**Figure 3:**
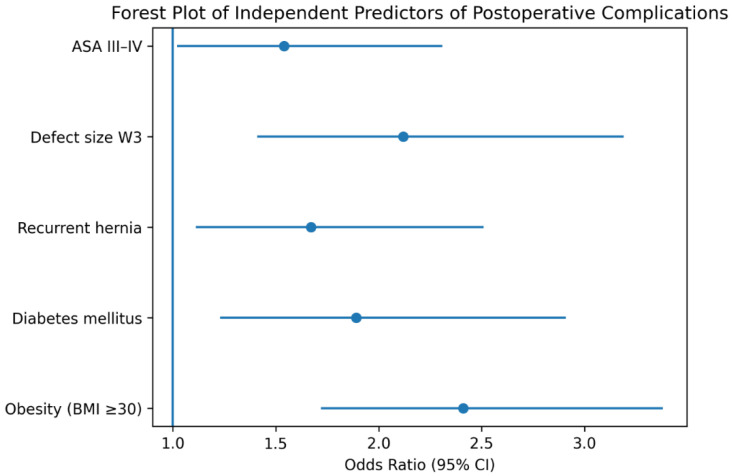
Forest plot illustrating the independent predictors of postoperative complications identified by multivariable logistic regression analysis. Obesity, diabetes mellitus, recurrent hernia, large defect size (W3), and higher ASA score were significantly associated with increased odds of complications.

Multivariable logistic regression analysis identified obesity, diabetes mellitus, recurrent hernia, large defect size (W3), and ASA III–IV classification as independent predictors of postoperative complications (Figure 3).

**Figure 4:**
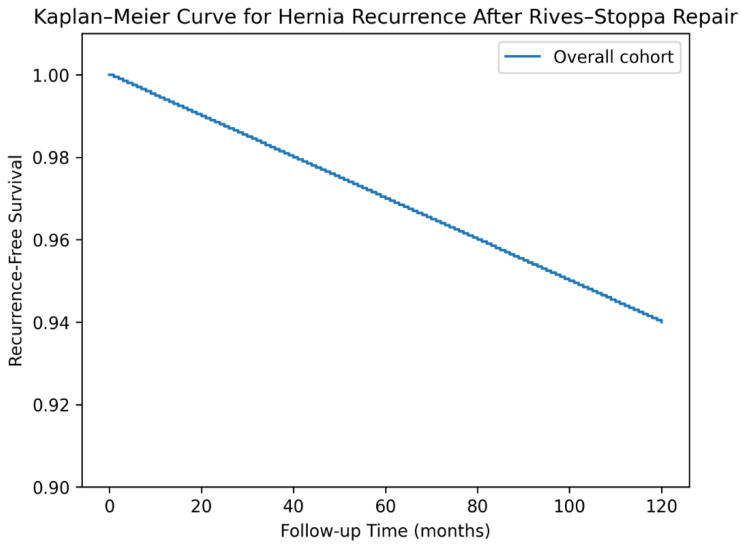
Kaplan–Meier curve illustrating recurrence-free survival following retromuscular Rives–Stoppa repair for incisional hernias during the follow-up period.

Kaplan–Meier analysis demonstrated a recurrence-free survival rate exceeding 90% during long-term follow-up, with an overall recurrence rate of approximately 6% (Figure 4).

Obesity (BMI ≥ 30) was significantly associated with postoperative complications (*p* < 0.001). Multivariable analysis identified obesity, diabetes, recurrent hernia, and large defect size as independent predictors.

## 4. Discussion

### 4.1. Principal Findings

The present study analyzed postoperative outcomes following retromuscular incisional hernia repair in a large cohort of 1262 patients. The overall postoperative complication rate was 19.5%, with seroma (10.5%) and surgical site infection (7.0%) representing the most frequent adverse events. Hernia recurrence occurred in 6% of patients during long-term follow-up, with Kaplan–Meier analysis demonstrating a recurrence-free survival exceeding 90%.

Multivariable analysis identified several independent predictors of postoperative complications, including obesity, diabetes mellitus, recurrent hernia, large defect size (W3), and higher ASA classification. Among these factors, obesity emerged as the strongest predictor of postoperative morbidity.

These findings highlight the multifactorial nature of postoperative outcomes after incisional hernia repair and confirm that both patient-related and hernia-related factors significantly influence surgical morbidity.

Importantly, the present study represents a large clinical cohort evaluating predictors of postoperative complications following retromuscular incisional hernia repair.

### 4.2. Retromuscular Repair and the Role of the Rives–Stoppa Technique

The Rives–Stoppa retromuscular repair remains one of the most widely accepted techniques for open incisional hernia repair due to its favorable biomechanical properties and relatively low recurrence rates. This technique involves reconstruction of the linea alba with placement of a mesh in the retrorectus plane, providing a well-vascularized environment that promotes mesh integration and reduces infection risk [18].

Compared with other mesh positions, the retromuscular plane allows wide mesh overlap and stable fixation through intra-abdominal pressure, facilitating tension-free abdominal wall reconstruction. Reported recurrence rates range from 1% to 15%, depending on patient characteristics and follow-up duration [19].

In the context of the present study, the observed recurrence rate of 6% and acceptable complication profile further support the reliability and durability of retromuscular mesh placement, particularly in complex incisional hernias.

### 4.3. Evolution of Minimally Invasive Retromuscular Approaches

Recent advances in abdominal wall surgery have aimed to reproduce the principles of retromuscular repair using minimally invasive approaches. The enhanced-view totally extraperitoneal (eTEP) technique represents one such development, combining the biomechanical advantages of retromuscular mesh placement with the benefits of minimally invasive surgery [14,19,20,21].

Early studies evaluating eTEP repair have reported promising outcomes, including low complication rates and acceptable recurrence rates, with some series demonstrating no recurrence during short-term follow-up [14,22]. Similarly, other laparo-endoscopic retromuscular techniques, such as eTEP-TAR, have shown favorable perioperative outcomes, including reduced surgical trauma and faster postoperative recovery [7].

However, these results should be interpreted with caution, as many minimally invasive studies include selected patient populations and shorter follow-up durations. In contrast, the present study reflects outcomes in a large, real-world cohort, including patients with more complex hernias. Therefore, while minimally invasive retromuscular approaches are promising, open retromuscular repair remains a reliable and widely applicable technique, particularly in complex cases.

### 4.4. Comparison of Postoperative Complications

In the present study, the overall complication rate was 19.5%, which is consistent with complication rates reported in previous abdominal wall reconstruction studies. Postoperative wound complications remain among the most frequent issues following ventral hernia repair, with seroma formation, surgical site infection, and hematoma representing the majority of postoperative adverse events.

Our findings are generally in line with previously published data evaluating retromuscular hernia repair. Studies analyzing robotic transabdominal retromuscular repair have reported lower complication rates, including seroma in approximately 2% of patients and hematoma in around 3% of cases [23,24]. Similarly, multicenter studies evaluating robotic retromuscular hernia repair have reported overall complication rates of approximately 7%, with recurrence rates around 2–3% during long-term follow-up [15,25].

The higher complication rate observed in the present study (19.5%) compared with rates reported in minimally invasive series (approximately 7–10%) may be explained by differences in patient selection and hernia complexity. Our cohort included a substantial proportion of patients with large defects (W3), recurrent hernias, and significant comorbidities, all of which are known to increase surgical risk. In contrast, many minimally invasive studies include more selected patient populations and less complex cases, which may contribute to the lower reported complication rates.

### 4.5. Hernia Recurrence

The recurrence rate of 6% observed in the present study is broadly consistent with outcomes reported in other retromuscular repair series. However, several recent studies evaluating robotic retromuscular repair have reported lower recurrence rates of approximately 2–4% at long-term follow-up and around 3% after three years [15,21,26].

These differences may be explained by variations in patient selection, hernia complexity, and follow-up duration. Minimally invasive studies often include more selected patient populations with smaller defects and fewer comorbidities, which may contribute to improved outcomes. In contrast, the present study reflects a real-world cohort that includes a substantial proportion of complex cases, such as large defects (W3) and recurrent hernias.

Therefore, the recurrence rate observed in this study should be interpreted within the context of patient heterogeneity and surgical complexity. These findings support the reliability and durability of open retromuscular repair, particularly in the management of complex incisional hernias.

### 4.6. Risk Factors for Postoperative Complications

In this study, obesity, diabetes mellitus, recurrent hernia, large defect size, and higher ASA classification were identified as independent predictors of postoperative complications. These findings are consistent with previous studies in abdominal wall reconstruction, which have highlighted the multifactorial nature of postoperative morbidity.

Obesity emerged as the strongest predictor of complications, in agreement with existing literature reporting an increased risk of surgical site occurrences in obese patients due to impaired wound healing, higher intra-abdominal pressure, and increased infection risk [27,28,29]. Similarly, diabetes mellitus was associated with increased postoperative complications, likely reflecting impaired immune response and delayed tissue repair, as consistently reported in prior studies [27].

Defect size and hernia recurrence also play a critical role in determining surgical outcomes. Larger defects (W3) require more extensive dissection and reconstruction, which may increase operative complexity and postoperative morbidity [30,31]. Recurrent hernias present additional technical challenges due to altered anatomy, fibrosis, and compromised tissue quality. These findings are in line with previous reports demonstrating worse outcomes in patients with complex or recurrent hernias.

Higher ASA classification was associated with an increased risk of postoperative complications, reflecting the overall burden of comorbidities and reduced physiological reserve. This observation is consistent with studies emphasizing the importance of preoperative risk stratification and optimization in high-risk surgical patients.

Chronic corticosteroid use represents a recognized risk factor for postoperative complications due to its immunosuppressive effects and its impact on wound healing. Although corticosteroid use was not specifically recorded in our retrospective database, its potential role in postoperative morbidity should be acknowledged. Additionally, important outcome measures such as ICU admission and postoperative mortality were not available in our dataset but may provide further insight into the severity of complications.

Recent studies have highlighted the role of novel inflammatory biomarkers, such as butyrylcholinesterase (BChE), in predicting postoperative complications. Reduced BChE levels have been associated with an increased risk of surgical site infections and more severe postoperative outcomes, particularly in colorectal surgery [32]. Although such biomarkers were not evaluated in the present study, their integration into future predictive models may improve risk stratification.

Smoking was associated with increased postoperative complications in univariate analysis, which is consistent with existing literature demonstrating impaired tissue oxygenation and delayed wound healing in smokers [33,34]. Although it was not retained as an independent predictor in the multivariable model, its clinical relevance remains significant, and preoperative smoking cessation should be encouraged.

Overall, these findings emphasize the importance of comprehensive preoperative assessment and optimization in patients undergoing incisional hernia repair, particularly in those with multiple risk factors.

### 4.7. Chronic Postoperative Pain

Chronic postoperative pain represents an important long-term complication following incisional hernia repair and may significantly impact patient quality of life, daily activities, and overall functional recovery. In the present study, chronic pain was reported in 3% of patients, which is relatively low compared with rates described in the literature. The development of chronic pain is multifactorial and may be related to nerve injury during surgical dissection, mesh-related inflammatory response, fixation techniques, and individual patient susceptibility.

In addition, factors such as preoperative pain, psychological status, and central sensitization mechanisms may contribute to the persistence of postoperative pain. The interaction between surgical trauma and patient-specific factors highlights the complexity of pain development following abdominal wall reconstruction [35].

Previous studies have reported chronic pain rates ranging from 5% to 20% after ventral hernia repair, depending on surgical technique, mesh type, and duration of follow-up. Retromuscular mesh placement has been associated with lower rates of chronic pain compared with other techniques, likely due to reduced nerve irritation, avoidance of direct contact with intra-abdominal structures, and more physiological mesh positioning [35,36].

Furthermore, advances in surgical techniques, including atraumatic dissection, selective use of mesh fixation, and nerve-sparing approaches, may contribute to reducing the incidence of chronic postoperative pain. These findings highlight the importance of meticulous surgical technique, appropriate mesh selection, and careful tissue handling in minimizing long-term postoperative pain and improving patient-reported outcomes [37].

### 4.8. Role of Mesh Type in Postoperative Outcomes

Mesh selection remains an important factor influencing surgical outcomes in abdominal wall reconstruction. However, recent studies comparing different polypropylene mesh types have demonstrated that retromuscular mesh placement provides comparable outcomes regardless of mesh structure, with no significant differences in recurrence or overall complication rates [5,38,39,40,41]. These findings suggest that mesh position may play a more critical role than mesh composition itself.

The biological response to implanted prosthetic mesh involves complex inflammatory and remodeling processes. Histopathological studies have shown that mesh implantation induces a foreign body granulomatous reaction characterized by multinucleated giant cells, chronic inflammatory infiltrates, and progressive fibrotic encapsulation of the prosthetic material [42]. These mechanisms reflect ongoing implant–tissue interaction and may contribute to postoperative complications and long-term tissue remodeling.

More recently, hybrid absorbable–permanent mesh devices designed for extraperitoneal placement have been introduced, aiming to combine structural support with reduced inflammatory response and improved tissue integration [1,43]. Although these technologies are promising, current evidence remains limited, and their long-term clinical benefits require further validation.

In the context of the present study, the use of a standardized retromuscular technique may have contributed to the relatively low recurrence rate and acceptable complication profile observed. Overall, these findings support the concept that optimal mesh positioning within the abdominal wall remains one of the most important determinants of surgical success, potentially outweighing differences in mesh material.

### 4.9. Clinical Implications

The identification of independent predictors of postoperative complications has important implications for clinical practice. The present findings support the concept of risk stratification in patients undergoing incisional hernia repair, as individuals with obesity, diabetes mellitus, or large hernia defects represent a particularly high-risk group for postoperative morbidity.

These results also highlight the importance of preoperative optimization strategies, including weight reduction, improved glycemic control, and smoking cessation, which may reduce the risk of postoperative complications and improve overall surgical outcomes.

Furthermore, the predictive model developed in this study demonstrated moderate discriminatory ability (AUC ≈ 0.73), suggesting that combinations of clinical variables can assist in identifying patients at increased risk. Such models may support individualized perioperative management, improve surgical planning, and facilitate more accurate patient counseling regarding expected risks and outcomes.

### 4.10. Strengths and Limitations

This study has several important strengths. The large cohort of 1262 patients represents a substantial clinical analysis evaluating postoperative complications after retromuscular incisional hernia repair. In addition, the use of multivariable logistic regression enabled the identification of independent predictors while adjusting for potential confounding variables. Furthermore, the inclusion of follow-up data with assessment of hernia recurrence provides additional insight into the durability of retromuscular repair.

Several limitations should be acknowledged. First, the retrospective design introduces the possibility of selection bias and unmeasured confounding factors. Second, this study reflects a single-center experience, which may limit the generalizability of the findings. Third, although recurrence was assessed, follow-up duration was not uniform across patients, and both the median follow-up time and loss to follow-up rate were not consistently available, which may have influenced recurrence detection.

Additionally, data regarding chronic corticosteroid use, ICU admission, and postoperative mortality were not available in our database and could not be included in the analysis. The predictive model was also not externally validated in an independent cohort, which may limit its applicability in different clinical settings. Furthermore, although all procedures were performed following a standardized surgical protocol, potential variability related to individual surgeon experience and intraoperative decision-making may have influenced postoperative outcomes. Finally, specific surgical details such as mesh fixation technique, use of component separation, and type of mesh were not systematically analyzed.

The findings of this study apply specifically to standard open retromuscular Rives–Stoppa repair and should not be extrapolated to procedures involving posterior component separation or TAR, which represent distinct surgical techniques with different technical complexity and risk profiles.

Future multicenter prospective studies are warranted to externally validate predictive models and to further refine risk stratification for postoperative complications in incisional hernia repair.

## 5. Conclusions

Retromuscular incisional hernia repair remains a safe and effective surgical technique associated with acceptable postoperative morbidity and relatively low recurrence rates. In this large cohort of 1262 patients, obesity, diabetes mellitus, recurrent hernia, large defect size, and higher ASA classification were identified as independent predictors of postoperative complications. These findings highlight the importance of patient-related and hernia-related factors in determining postoperative outcomes. Incorporating these variables into clinical risk stratification may support preoperative optimization strategies and improve perioperative management in patients undergoing abdominal wall reconstruction.

## Figures and Tables

**Table 1:** Baseline Characteristics of the Study Population.

Variable	Total (N = 1262)
**Age (years), mean ± SD**	61.5 ± 12.4
**Female sex, n (%)**	694 (55%)
**Male sex, n (%)**	568 (45%)
**Body mass index (kg/m^2^), mean ± SD**	31.2 ± 5.8
**Normal weight (BMI < 25), n (%)**	189 (15%)
**Overweight (BMI 25–29.9), n (%)**	416 (33%)
**Obesity (BMI ≥ 30), n (%)**	657 (52%)
**Diabetes mellitus, n (%)**	278 (22%)
**Active or recent smoking, n (%)**	252 (20%)
**Hypertension, n (%)**	782 (62%)
**Pulmonary disease (COPD/asthma), n (%)**	139 (11%)
**Recurrent hernia at presentation, n (%)**	290 (23%)
**ASA I–II, n (%)**	820 (65%)
**ASA III–IV, n (%)**	442 (35%)

**Table 2:** Hernia Characteristics and Operative Data.

Variable	n (%) or Mean ± SD
**Midline hernia**	1073 (85%)
**Lateral hernia**	189 (15%)
**Defect size W1 (<4 cm)**	315 (25%)
**Defect size W2 (4–10 cm)**	694 (55%)
**Defect size W3 (>10 cm)**	253 (20%)
**Operative time (minutes)**	135 ± 35
**Length of hospital stay (days)**	7.5 ± 3.2

**Table 3:** Postoperative Complications.

Complication	n (%)
**Any complication**	246 (19.5%)
**Seroma**	132 (10.5%)
**Surgical site infection (SSI)**	88 (7.0%)
**Hematoma**	44 (3.5%)
**Wound dehiscence**	19 (1.5%)
**Mesh infection**	19 (1.5%)
**Enterocutaneous fistula**	6 (0.5%)
**Chronic pain (>3 months)**	38 (3.0%)
**Hernia recurrence**	76 (6.0%)

**Table 4:** Univariate Analysis of Risk Factors for Postoperative Complications.

Variable	Complication (%)	No Complication (%)	*p*-Value
**Obesity (BMI ≥ 30)**	29%	71%	<0.001
**Diabetes mellitus**	31%	69%	0.012
**Smoking**	27%	73%	0.041
**Recurrent hernia**	33%	67%	0.008
**Defect size W3**	36%	64%	<0.001
**ASA III–IV**	34%	66%	0.015

**Table 5:** Multivariable Logistic Regression for Predictors of Complications.

Variable	Odds Ratio (OR)	95% CI	*p*-Value
**Obesity (BMI ≥ 30)**	2.41	1.72–3.38	<0.001
**Diabetes mellitus**	1.89	1.23–2.91	0.004
**Recurrent hernia**	1.67	1.11–2.51	0.013
**Defect size W3**	2.12	1.41–3.19	<0.001
**ASA III–IV**	1.54	1.02–2.31	0.038

## Data Availability

The data presented in this study is available on request from the corresponding author. The data is not publicly available due to patient confidentiality.

## Abbreviations

ASA	American Society of Anesthesiologists
BMI	Body Mass Index
CI	Confidence Interval
COPD	Chronic Obstructive Pulmonary Disease
EHS	European Hernia Society
OR	Odds Ratio
ROC	Receiver Operating Characteristic
SSI	Surgical Site Infection
TAR	Transversus Abdominis Release
AUC	Area Under the Curve
